# Fatal Thrombosis of a Flow Diverter due to Ibuprofen-related Antagonization of Acetylsalicylic Acid

**DOI:** 10.1007/s00062-015-0487-7

**Published:** 2015-12-02

**Authors:** R. Martinez-Moreno, M. Aguilar, C. Wendl, H. Bäzner, O. Ganslandt, H. Henkes

**Affiliations:** 1 Neuroradiological Clinic, Katharinenhospital, Klinikum Stuttgart, Kriegsbergstraße 60, 70174 Stuttgart, Germany; 2 Neurological Clinic, Klinikum Stuttgart, Stuttgart, Germany; 3 Neurosurgical Clinic, Klinikum Stuttgart, Stuttgart, Germany; 4 Medical Faculty, University of Duisburg-Essen, Essen, Germany

## Introduction

The use of flow diverters (FD) became well established for the treatment of fusiform and selected sidewall intracranial aneurysms [1]. Even though flow diversion is relatively safe and efficacious, it is not without potential complications. They include ischemic and hemorrhagic events [2]. One of the critical aspects of this technique is the required pre- and postprocedural dual platelet function inhibition. The postprocedural medication protocol following a FD implantation has two main goals: achieving an adequate level of platelet activity inhibition and reducing the inflammatory reaction in the aneurysmal wall [3, 4]. Therefore, we included in our protocol the dual antiplatelet therapy (in this case aspirin and ticagrelor) combined with ibuprofen and steroids to mitigate thrombus-related inflammation. A potential interference of ibuprofen with the antiaggregation effect of aspirin has been described [5]. Given the widespread use of these two drugs, this pharmacological interaction should be taken into account after an intracranial FD implantation.

## Case Report

**Fig. 1:**
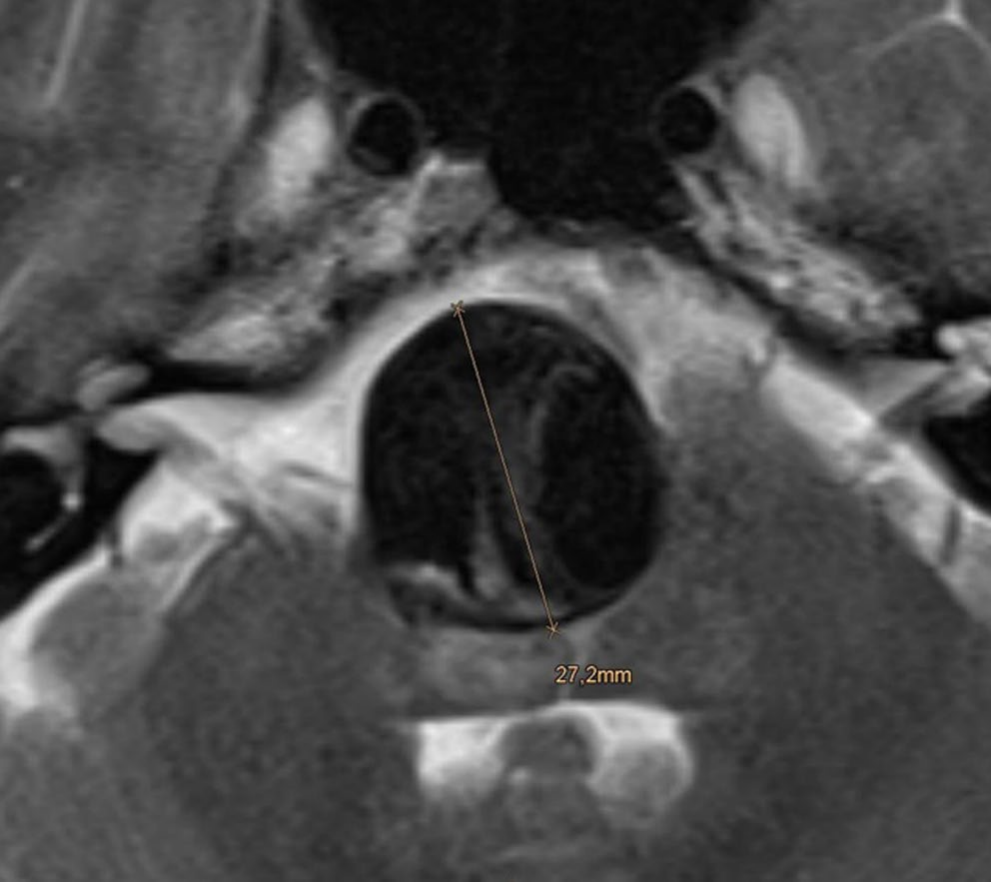
T2-weighted axial magnetic resonance imaging (MRI) of a wide necked partially thrombosed proximal basilar artery aneurysm, prior to treatment

**Fig. 2:**
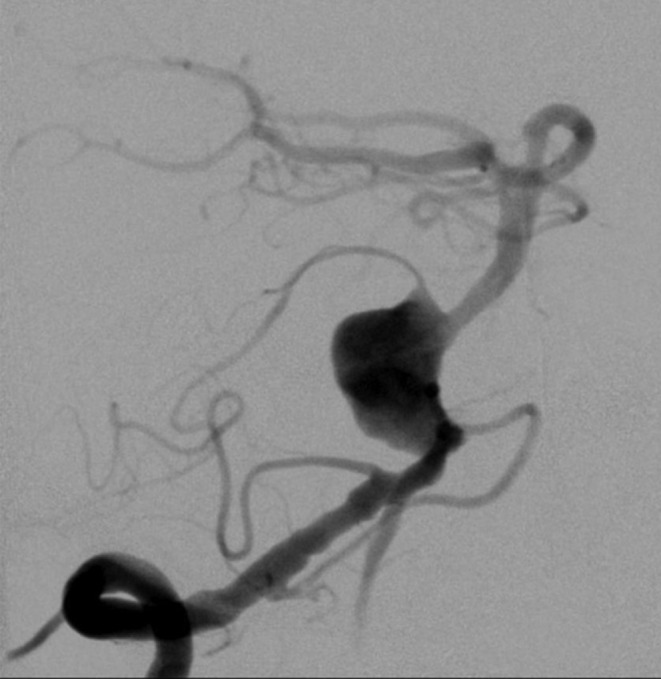
Digital subtraction angiography (DSA) image of the aneurysm, showing the very wide neck of the aneurysm. The right anterior inferior cerebellar artery (AICA) is integrated in the aneurismal sac

A 53-year-old man was referred to us because of an incidental, large, wide neck and partially thrombosed saccular aneurysm of the proximal basilar trunk. The evaluation and comparison of magnetic resonance imaging (MRI) findings showed an increase in diameter of the aneurysm within 7 months and a moderate mass effect on the brain stem. The largest axial diameter at the time of endovascular treatment was 27 mm (Fig. 1). As seen in the angiography prior to the treatment, the origin of the right anterior inferior cerebellar artery (AICA) was integrated in the aneurysm sac (Fig. 2).

### Treatment

**Fig. 3:**
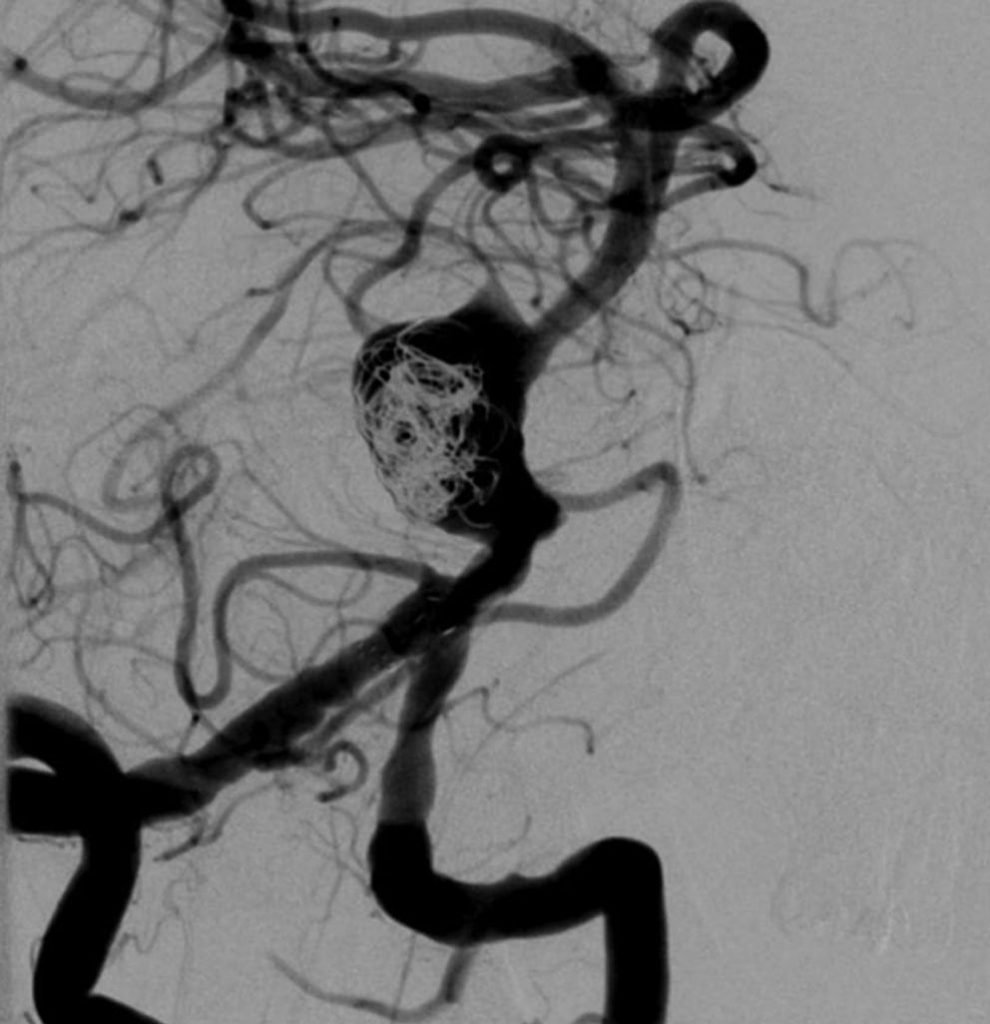
Digital subtraction angiography (DSA) image after partial coil occlusion and reconstruction of the parent vessel with an Enterprise2 stent and two p64 flow diverters

The treatment started with the surgical placement of a ventriculo-peritoneal shunt to prevent a potential cerebrospinal fluid circulation disturbance. Seven days later, the basilar artery was reconstructed through a combination of a conventional stent (Enterprise2, 4/39 mm, Codman Neurovascular) and two FDs (p64, 2 × 4/24 mm, Phenox). The Enterprise2 stent was used as a scaffold to provide support for the flow modulating implants. Additionally, five coils were implanted in the caudal compartment of the aneurysmal sac (Fig. 3) to enhance the flow diverting effect of the two p64. Immediately prior to the intervention the patient received a loading dose of 500 mg ASA and 180 mg ticagrelor per os. Platelet function inhibition was confirmed before the treatment (Multiplate Test), being the values (AUC) adequately under the response threshold. The treatment was performed under anticoagulation (3000 units of heparin IV) and to ensure a correct platelet inhibition, a body weight adapted bolus of 15.8 mg eptifibatide was given. To prevent perianeurysmal edema and excessive inflammation, 40 mg of dexamethasone were given intravenously at the end of the procedure. Our postprocedural medication protocol of dual antiaggregation (100 mg ASA and 2 × 90 mg ticagrelor, both daily) is combined with steroid therapy for 3 days (3 × 4 mg dexamethasone daily) and 1 × 400 mg ibuprofen daily for 3 weeks (per os, taken at least 2 h *after* ASA).

### Outcome, Follow-up

**Fig. 4:**
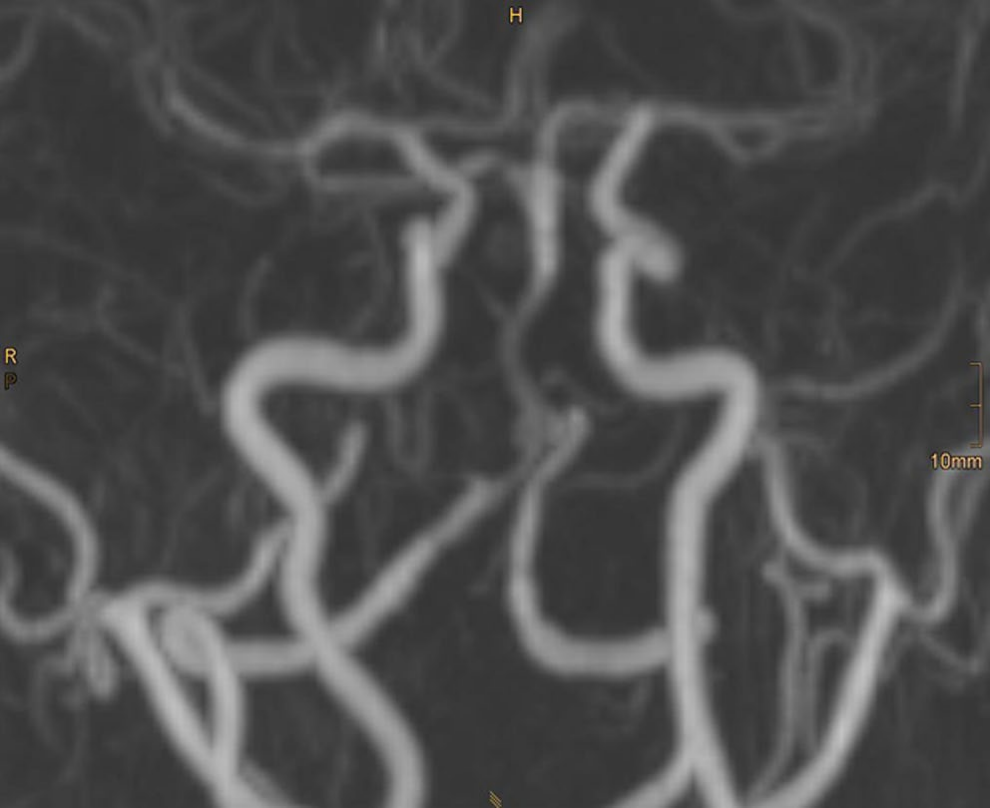
Time-of-flight magnetic resonance angiography (TOF-MRA) 3 days later confirms the patency of the basilar artery and the right anterior inferior cerebellar artery (AICA)

The MRI/MRA before discharge confirmed patency of the vertebral and basilar arteries, including the right AICA, and thrombosis of the aneurysmal sac (Fig. 4).

**Fig. 5:**
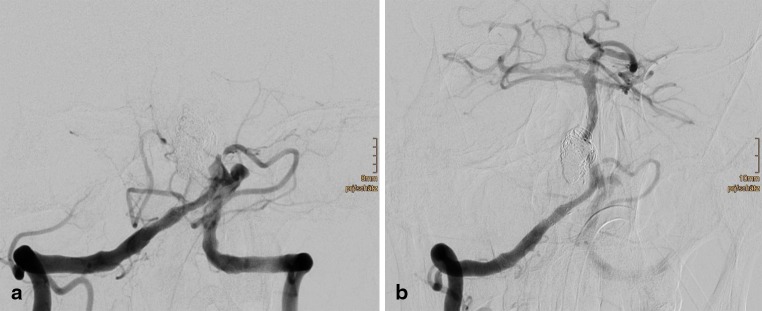
Digital subtraction angiography (DSA) 1 week later confirms acute thrombotic occlusion of the flow diverters (**a**), which were recanalized with aspiration thrombectomy (**b**)

**Fig. 6:**
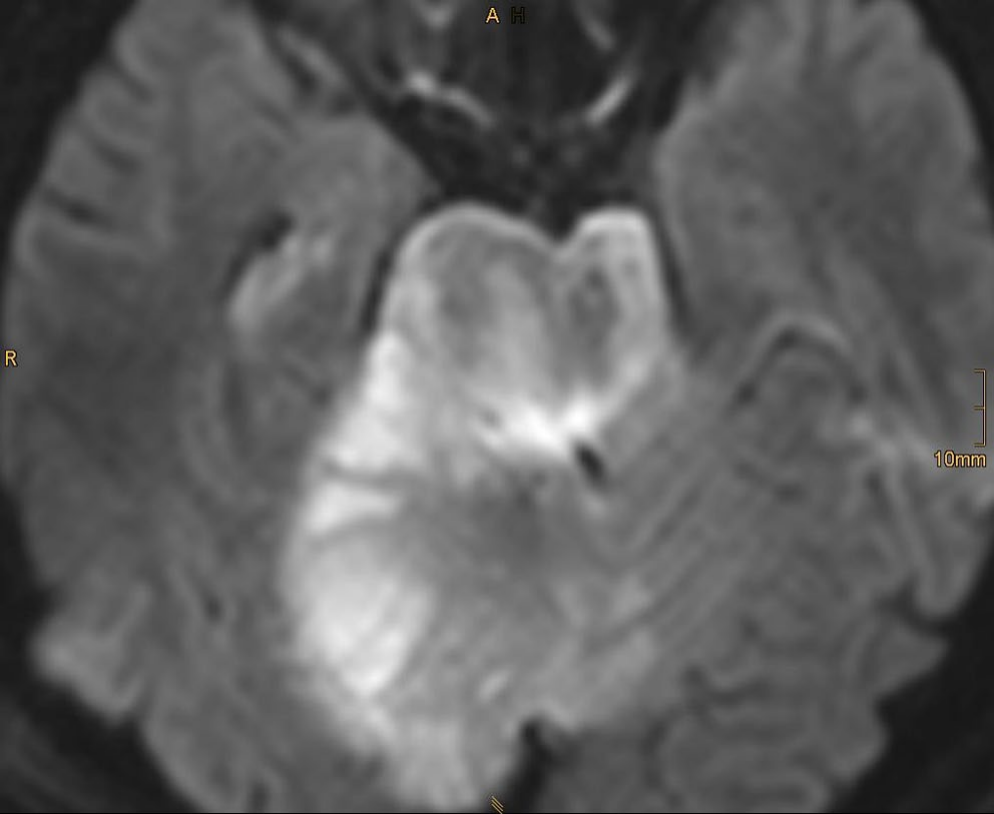
Subsequent magnetic resonance imaging (MRI), however, showed massive brain stem infarction

One week later the patient collapsed at home. He experienced progressive coma during an extended transfer to our facility. A MRI examination upon arrival revealed complete occlusion of the basilar artery. The family confirmed that all prescribed medication had been taken; however, aspirin and ibuprofen were taken together. A Multiplate test revealed that there was no aspirin effect on the thrombocytic function. An analysis of urine and venous blood confirmed the presence of ibuprofen in both. An aspiration thrombectomy was immediately performed, and the basilar artery was successfully recanalized (Fig. 5a, b). On MRI, extensive ischemic damage of the pons and the right AICA territory was found post thrombectomy (Fig. 6). Increasing edema and tonsillar herniation resulted in death 4 days later due to cardiorespiratory arrest.

## Discussion

Aneurysms of the basilar artery are rare. Basilar trunk aneurysms are classified as segmental ectasia without intraluminal thrombus or as dissecting aneurysms with widespread disruption of the elastic lamina, thickened intima and extensive intraluminal thrombus [6]. Some of these segmental diseases result in aneurysms with a predominant saccular component, as in our case. These aneurysms of the basilar trunk, if left untreated, have poor survival rates. Patients suffer from progressive brain stem compression or subarachnoid hemorrhage, or both.

Reconstructive treatment of wide neck and fusiform vertebrobasilar aneurysms has been facilitated with the advent of FDs. The prognostic impact of a reconstructive endovascular treatment of large and giant dolichoectatic and dissecting vertebrobasilar aneurysms remains difficult to determine, but given the poor prognosis, treatment should be considered in symptomatic patients and in growing aneurysms. The goal of the procedure described above would not only be exclusion of the aneurysm from the normal blood circulation, but also to provide a structural support for the artery and facilitate the development of a neointimal layer [6]. This structure could be a combination of conventional stents and/or FDs, coaxially implanted and covering the pathologic segment of the basilar trunk.

An accumulating body of evidence implicates inflammation as a critical contributor to aneurysm formation, progression and eventual rupture [7, 8]. The ESMINT Retrospective Analysis of Delayed Aneurysm Ruptures after Flow Diversion (RADAR) showed that the vast majority of the aneurysms with delayed rupture were very large or giant (mean diameter of 24 mm) [9]. Another alarm sign could be recent growth, indicating instability of the aneurysm wall. Both, large size and recent growth were present in our patient. It is described that the acute formation of massive clot inside an aneurysm after flow diversion can act as a trigger for aggressive inflammatory processes in the aneurysmal wall, leading to a complete degeneration of the wall and consequently causing delayed rupture in approximately 1–2 % of cases [3, 4, 9]. The rapid blood clot formation inside an aneurysm after FD implantation would be the key factor in the overwhelming of the defense mechanism of vessel remodeling against autolytic activities. Histopathologically this clot has been identified as a disorganized thrombus without significant cellular colonization. On the other hand, the intraaneurysmal thrombosis induced by platinum and tungsten coils has demonstrated to promote the formation of a granulation tissue with capillary in growth as well as the proliferation and progressive replacement of the blood clot with increasingly collagenized fibrous scar tissue [10]. Therefore, a combined strategy of flow diversion and partial coil-occlusion of the aneurysmal sac may be used in order to stimulate a stable thrombosis of the aneurysm.

The second part of the strategy to control the inflammatory processes in the aneurysm sac includes the combination of dual antiplatelet medication (aspirin and clopidogrel or ticagrelor) with an additional nonsteroidal anti-inflammatory drug (NSAID) and steroids immediately after the intervention.

The interaction of various groups of NSAIDs with the thrombocyte inhibitory effect of aspirin has been relatively well described in the past, and recently analyzed in a placebo-controlled, ex vivo, serial placebo-controlled crossover study, among others [5, 11]. Inflammatory processes leading to nociceptive pain and thrombocyte aggregation are both mediated by different isoforms of the same enzyme, the cyclooxygenase (COX). COX-1 plays a major role in thrombocyte aggregation; COX-2 participates in the inflammatory response to tissue damage. In this study ibuprofen and naproxen, nonselective agents with an inherent weak thrombocyte inhibitory action, inhibit aspirin’s antiaggregant effect significantly, whereas the COX-2 selective NSAIDs meloxicam and etoricoxib caused no significant aspirin inhibition.

The effect of Ibuprofen administration in patients under aspirin therapy was also well described in a publication from Catella-Lawson et al. [11, 12]. According to their results Ibuprofen might competitively bloc the inhibition of platelet COX-1 activity achieved by ASA. However, a single dose of Ibuprofen given 2 h *after* the administration of ASA could avoid this interaction. The authors warn, otherwise, that in a second study simulating a more clinically relevant ibuprofen dosage regimen (three times daily) the time gap of 2 h between the ASA and Ibuprofen morning doses failed to bypass the interaction.

Our case illustrates the potentially dramatic consequences of this pharmacological interaction and forces one to have these results in mind to avoid fatal thromboembolic complications in the future.

## Conclusion

Given their poor prognosis, the symptomatic, wide neck or fusiform, dolichoectatic or dissecting, vertebrobasilar aneurysms should be treated. A reconstructive endovascular therapy with FDs either combined with conventional stents or alone is feasible in many cases. In large or giant aneurysms, the use of coils to stabilize the clot formation is recommended. The postprocedural combination of dual antiplatelet medication with anti-inflammatory agents such as steroids and selective COX-2 inhibitors is also needed. The combination of aspirin and nonselective NSAIDs such as ibuprofen or naproxen must be avoided.
